# Connexin 26 Expression in Mammalian Cardiomyocytes

**DOI:** 10.1038/s41598-018-32405-2

**Published:** 2018-09-18

**Authors:** S. Moscato, M. Cabiati, F. Bianchi, F. Vaglini, M. A. Morales, S. Burchielli, L. Botta, A. R. M. Sabbatini, A. Falleni, S. Del Ry, L. Mattii

**Affiliations:** 1 Department of Clinical and Experimental Medicine, Unit of Histology, University of Pisa, Pisa, Italy; 2 Biochemistry and Molecular Biology Laboratory, Institute of Clinical Physiology, CNR, Pisa, Italy; 3 Department of Translational Research and of New Surgical and Medical Technologies, University of Pisa, Pisa, Italy; 4 G. Monasterio Foundation, Pisa, Italy; 5 Department of Cardiac Surgery, Niguarda Ca’ Granda Hospital, Milan, Italy; 6 Department of Surgical, Medical and Molecular Pathology and of Emergency Medicine, University of Pisa, Pisa, Italy

## Abstract

Connexins are a family of membrane-spanning proteins named according to their molecular weight. They are known to form membrane channels mediating cell-cell communication, which play an essential role in the propagation of electrical activity in the heart. Cx26 has been described in a number of tissues but not in the heart, and its mutations are frequently associated with deafness and skin diseases. The aim of this study was to assess the possible Cx26 expression in heart tissues of different mammalian species and to demonstrate its localization at level of cardiomyocytes. Samples of pig, human and rat heart and H9c2 cells were used for our research. Immunohistochemical and molecular biology techniques were employed to test the expression of Cx26. Interestingly, this connexin was found in cardiomyocytes, at level of clusters scattered over the cell cytoplasm but not at level of the intercalated discs where the other cardiac connexins are usually located. Furthermore, the expression of Cx26 in H9c2 myoblast cells increased when they were differentiated into cardiac-like phenotype. To our knowledge, the expression of Cx26 in pig, human and rat has been demonstrated for the first time in the present paper.

## Introduction

Connexins (Cxs) form membrane channels which play an essential role in the propagation of electrical activity throughout the heart. Their dysfunction has been linked to congenital malformations of heart and to a wide variety of cardiac pathologies^1^. Over twenty isoforms of Cxs have been recognized in mammals and classified according to their molecular weight. So far, seven isoforms, namely Cx30.2, Cx37, Cx40, Cx43, Cx45, Cx46 and Cx57, have been reported to be expressed in cardiac myocytes^1,2^. A space defined pattern of expression of cardiac Cxs correlates with functional differentiation within the heart. For example, Cx43, the major isoform in heart, is present in working ventricular and atrial myocardium. Cx40 and Cx45 are mostly concentrated in the conduction system, while Cx40 is abundantly expressed in atrial myocytes^2^.

In heart, six of the transmembrane Cxs form hemichannels called connexons. They are generally located at the intercalated discs and can align in adjacent cells to create gap junctions that allow the intercellular exchange of 1–1.8 kDa molecules such as electrical signals, small metabolites and second messengers. However, recent evidence suggests that Cx43 is localized also outside the gap junctions, where it represents a part of a protein interacting network, the connexome, involved in the propagation of an excitatory current between adjacent cells^3^. Moreover, Cxs may have non-canonical function also in other tissues since they may be organized in free connexons at plasma membrane level, joining intracellular and extracellular compartments. They may also be individually involved in the modulation of cell proliferation and tumor progression, interacting with intracellular proteins such as oncogene products, protein kinases or cytoskeleton elements^4,5^.

Cx26 has been described in a number of tissues but not in the heart and its mutations are frequently associated with deafness and skin diseases^6,7^. An altered expression of Cx26 in colonic smooth muscle cells may predispose the formation of diverticular lesions^8^ while the reduced expression of Cx26 may contribute to the low sensitivity of hepatocellular carcinoma towards the chemotherapeutic agent oxaliplatin^9^.

Due to the lack of information in the literature about the presence of this connexin at cardiac level, the aim of this study was to investigate the expression of Cx26 in the heart of different mammalian species. Therefore, we tested Cx26 expression in pig, human, and rat heart tissues and in a rat cardiomyocyte cell line by using different approaches including both immunohistochemistry and molecular biology techniques to evaluate its localization in myocardial cells.

## Results

All the results shown in this paper were obtained from at least 3 repeated experiments.

### Cx26-mRNA is expressed in pig, human and rat heart

**Figure 1:**
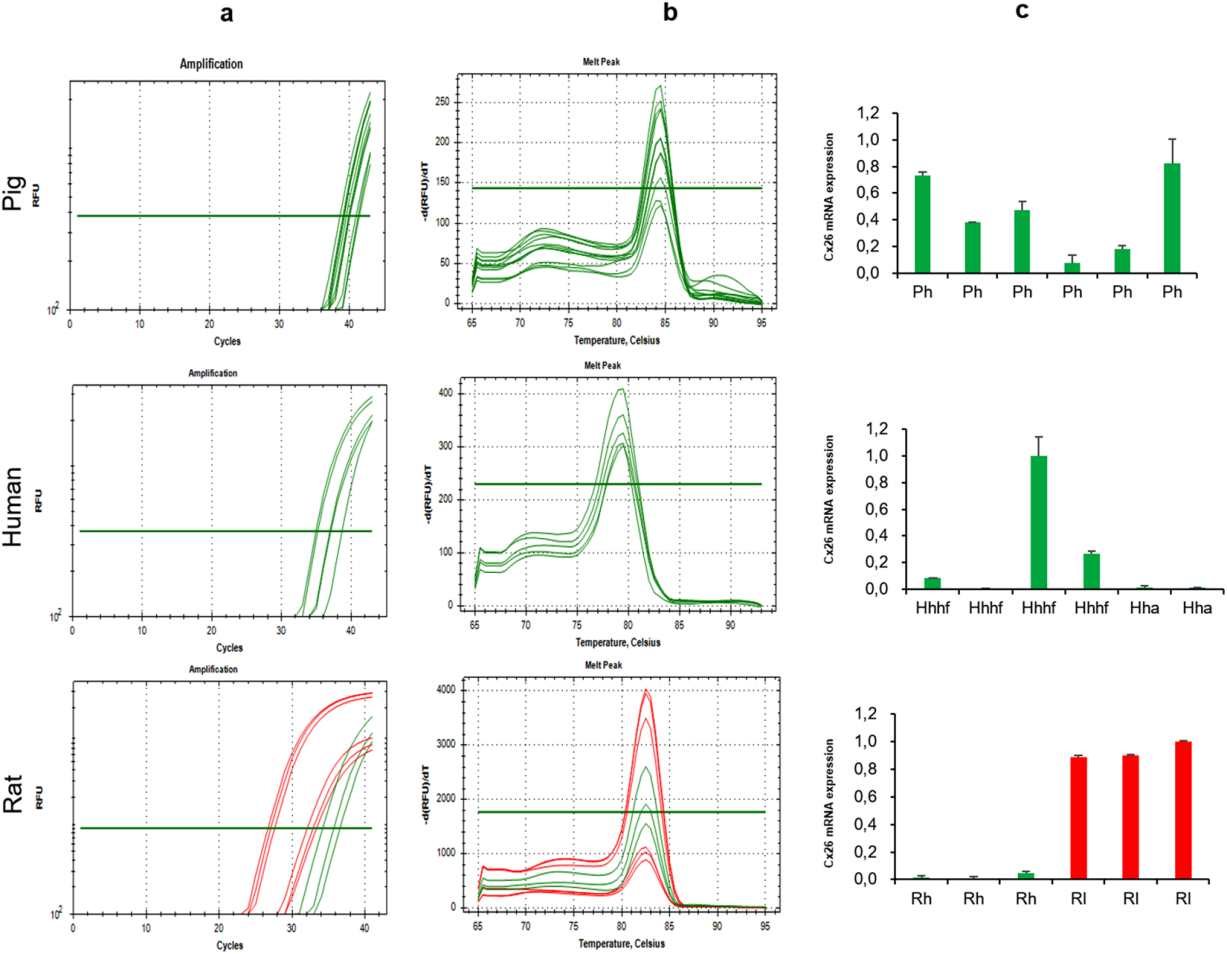
Cx26-mRNA is expressed in pig, human, and rat heart. Results of RT-PCR performed on heart samples (green) of pig left ventricle (Ph), human auricle (Hha)/ventricle with failure (Hhhf), rat (Rh) and liver tissue samples (red) of rat (Rl). (**a**) Example of threshold cycle (Ct) and relative amplification curves (**b**) Melting peak (negative first derivative of the change in fluorescence plotted as a function of temperature). (**c**) Absolute quantity of Cx26.

Real-Time PCR experiments allowed to highlight Cx26-mRNA expression in pig, human and rat heart samples (Fig. 1). It has been possible to obtain specific threshold cycles and relative amplification curves for each species. To optimize the thermocycling profile of each reference gene, optimal annealing temperature and RNA concentration were assessed for each designed PCR primer. RT-PCR analysis efficiency resulted in the range of 95–105% and with a linear standard curve, R^2^, greater than |0.990| Rat livers, used as positive controls^10^, expressed Cx26-mRNA. In Fig. 1c, absolute quantity of Cx26-mRNA in LV pig heart samples, in human heart samples (collected from patients with different grade of heart failure and from auricle), as well as in rat heart and liver samples, is reported. These values were quite different between the heart samples compared to the liver samples (positive tissue control), that were constantly higher.

### Cx26-protein is expressed in pig, human and rat heart

**Figure 2:**
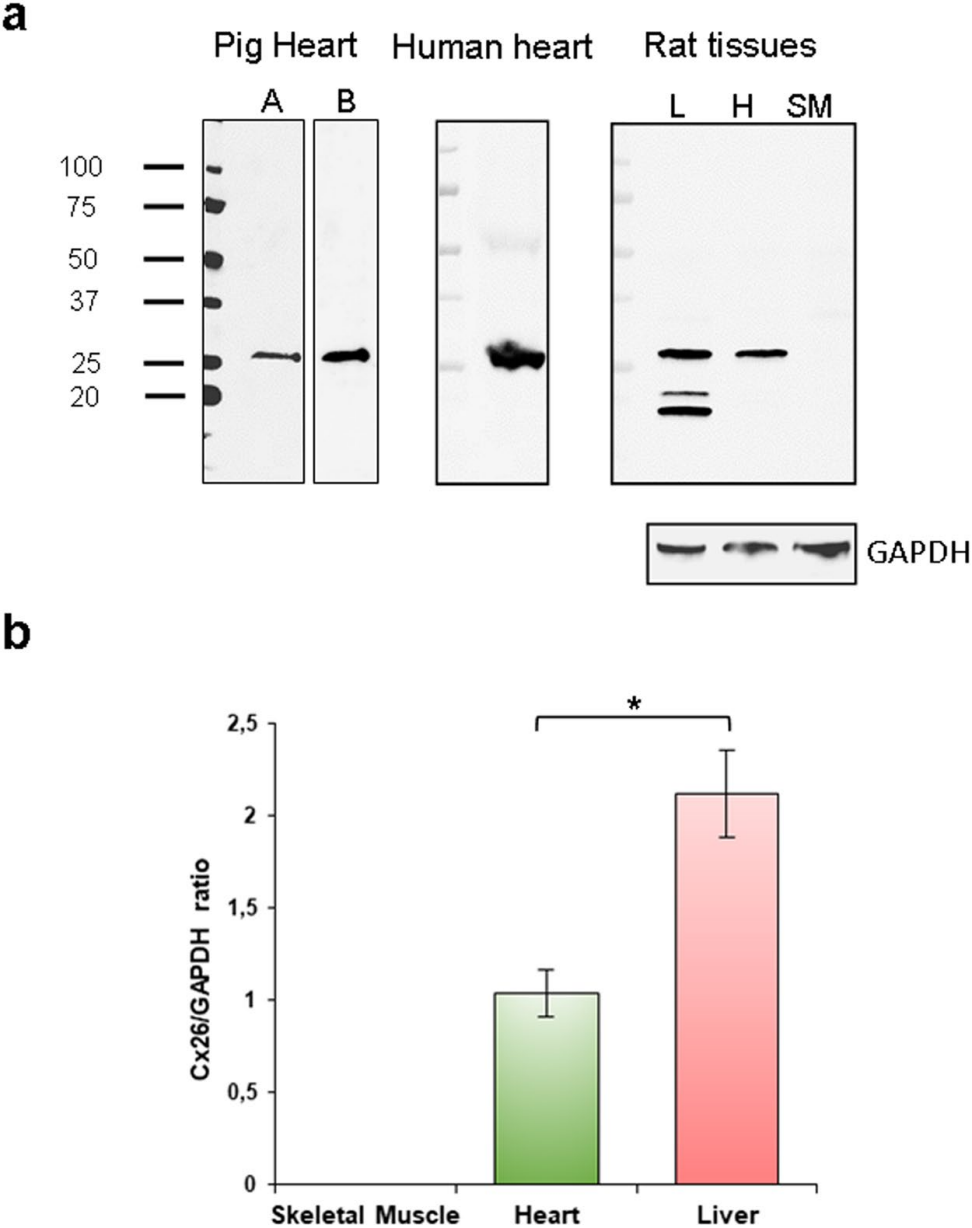
Western blotting analysis detects Cx26 using RαCx26-Ct or RαCx26-cl antibodies. (**a**) RαCx26-Ct (A) and RαCx26-cl (B) antibodies detected a specific 26 kDa band on pig heart tissue lysates (lanes A and B are cropped blots obtained from the same gel). RαCx26-cl recognized the 26 kDa band on human and rat heart (H) lysates and on rat liver (L) sample (positive control) although two unspecific bands at molecular weights lower than 26 kDa were detected. No bands were detected on smooth muscle (SM) tissue lysates (negative control) by RαCx26-cl. RαGAPDH was used as protein loading control for rat tissue samples run on the same gel. All gels were run in the same experimental conditions and blots had similar exposure times (see material and methods for details). Images are representative of one sample of the three for each species. (**b**) The histogram represents the Cx26 protein expression in rat tissues. Data are expressed as mean ± standard error of mean (SEM). *p < 0.005

**Figure 3:**
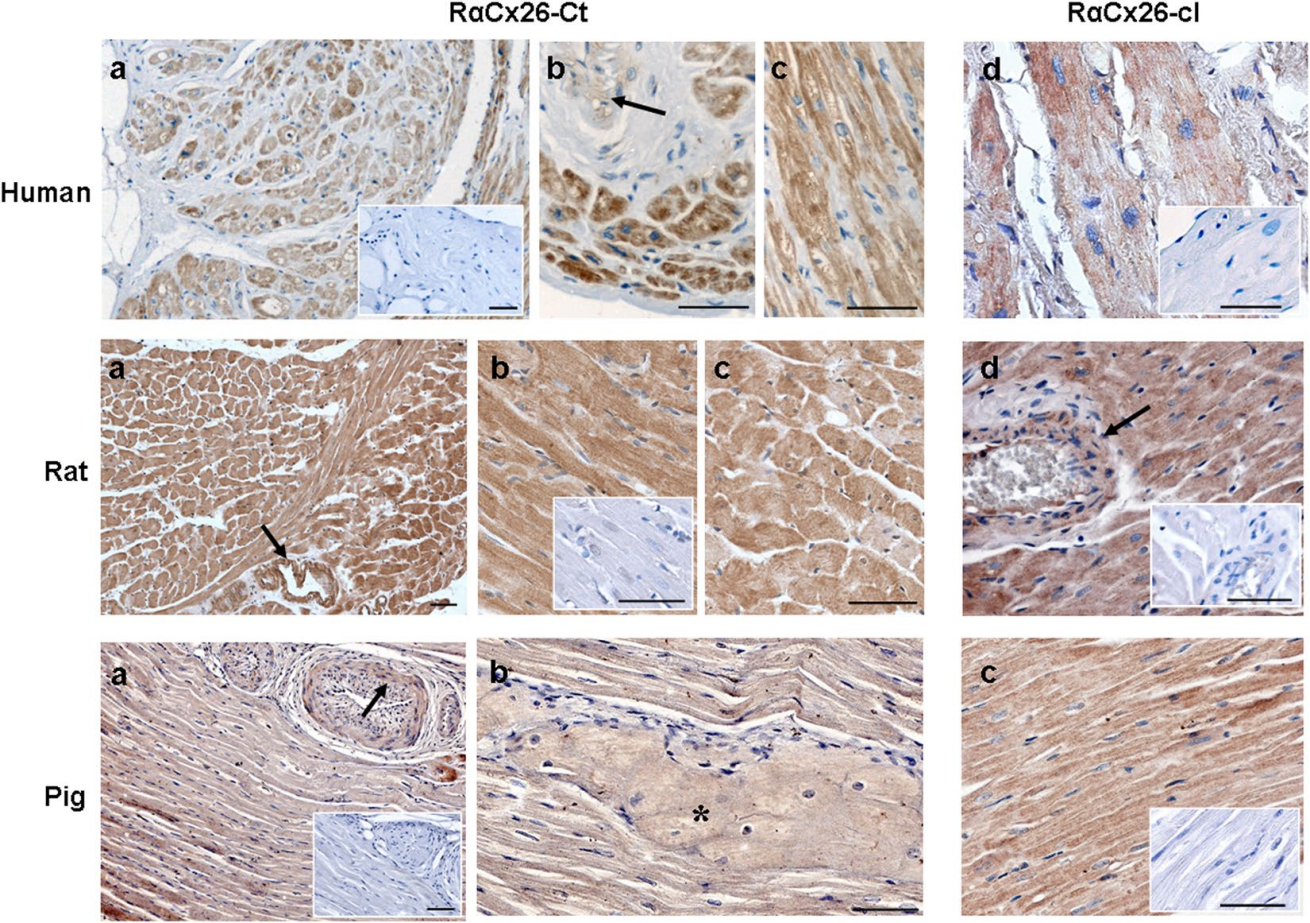
Human, rat and pig cardiomyocytes immunoreact with RαCx26 antibodies. Representative images of immunohistochemical analysis for Cx26 by RαCx26-Ct or RαCx26-cl antibodies. Human: (**a**) immunoperoxidase shows brown immunoreaction in the cell cytoplasm of auricle cardiomyocytes; higher magnification of cross (**b**) and longitudinal (**c** and **d**) sections showing positivity in cardiomyocytes and in smooth muscle cells of vessel (arrow); Rat: (**a**) and (**d**) immunoperoxidase shows brown immunoreaction in the cell cytoplasm of working cardiomyocytes and in smooth muscle cells of vessels (arrows); positivity of cardiomyocytes and smooth muscle cells has shown, at higher magnification, in longitudinal (**b** and **d**) and cross sections (**c**); Pig: (**a)** immunoperoxidase shows brown immunoreaction in the cell cytoplasm of working cardiomyocytes and in smooth muscle cells of vessels (arrow); higher magnification shows the positivity of cardiomyocytes of the conduction system (**b**, *) and of working cardiomyocytes in longitudinal section (**c**).Negative controls are showen in the squares. Scale bars: 50 µm.

**Figure 4:**
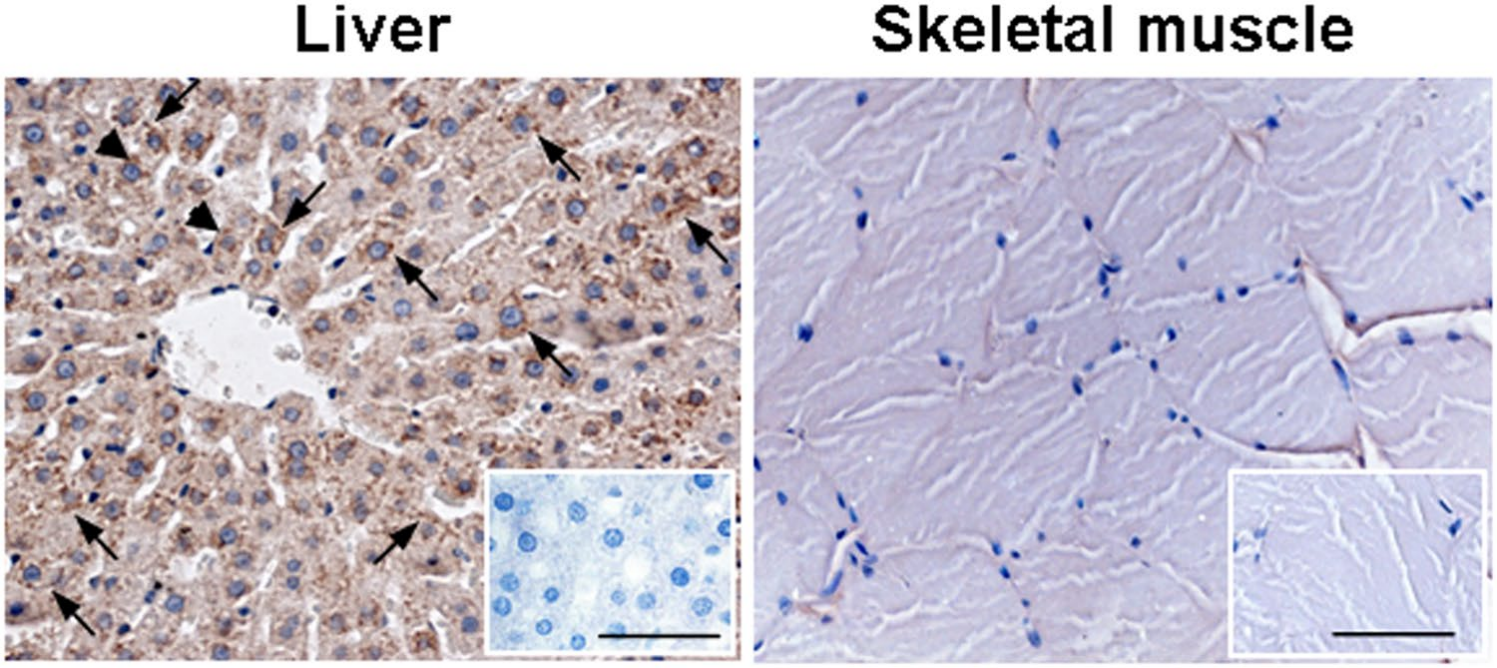
Positive and negative tissue controls for Cx26. Representative images of immunohistochemical analysis for Cx26 on rat liver and skeletal muscle sections by RαCx26-cl antibody. Cx26 positive brown punctate staining of hepatocyte plasma membrane (arrows) and cytoplasm (arrowheads) is shown in liver. By contrast, Cx26 staining is absent in skeletal muscle. Negative controls are showen in the squares. Scale bars: 50 µm.

We evaluated the Cx26 protein expression on tissue sections of pig, human and rat heart by immunohistochemistry. To do this, we performed the experiments using two Cx26-primary antibodies, RαCx26-Ct and RαCx26-cl, whose specificity was verified by both western blotting (Fig. 2a) and immunoperoxidase on positive and negative tissue controls (Fig. 3 and 4). Namely, RαCx26-Ct was validated by western blot on pig heart tissue. Indeed, the antibody recognized a single band at 26 kDa in all the pig tissue tested samples (=3) (Fig. 2a). Even RαCx26-cl was validated by western blot on in pig, human and rat heart tissues. This antibody also detected a single band at 26 kDa in all the samples (=3 for any species). Moreover, RαCx26-cl detected the 26 kDa band in protein samples of rat liver (positive control^10^) while it did not react with rat skeletal muscle samples (negative tissue control^11^) even though the protein normalization was verified by anti-GAPDH antibody reaction (Fig. 2a). Only on rat liver samples, RαCx26-cl detected two unusual additional bands at lower molecular weight than 26 kDa. Despite this fact, the band of 26 kDa was detected in liver protein lysate and it was compared to the specific one resulting in rat heart sample. As shown in Fig. 2b the quantity of Cx26 protein in liver samples was significantly higher than in heart samples.

In immunoperoxidase analysis, the two RαCx26 antibodies positively marked vessel smooth muscle cells (internal positive control tissue^8,12^) in pig, human and rat hearts (arrows in Fig. 3). Moreover, RαCx26-cl showed an immunopositive reaction in rat hepatocytes but not on rat skeletal muscle fibers that were used as positive and negative tissue controls, respectively (Fig. 4).

**Figure 5:**
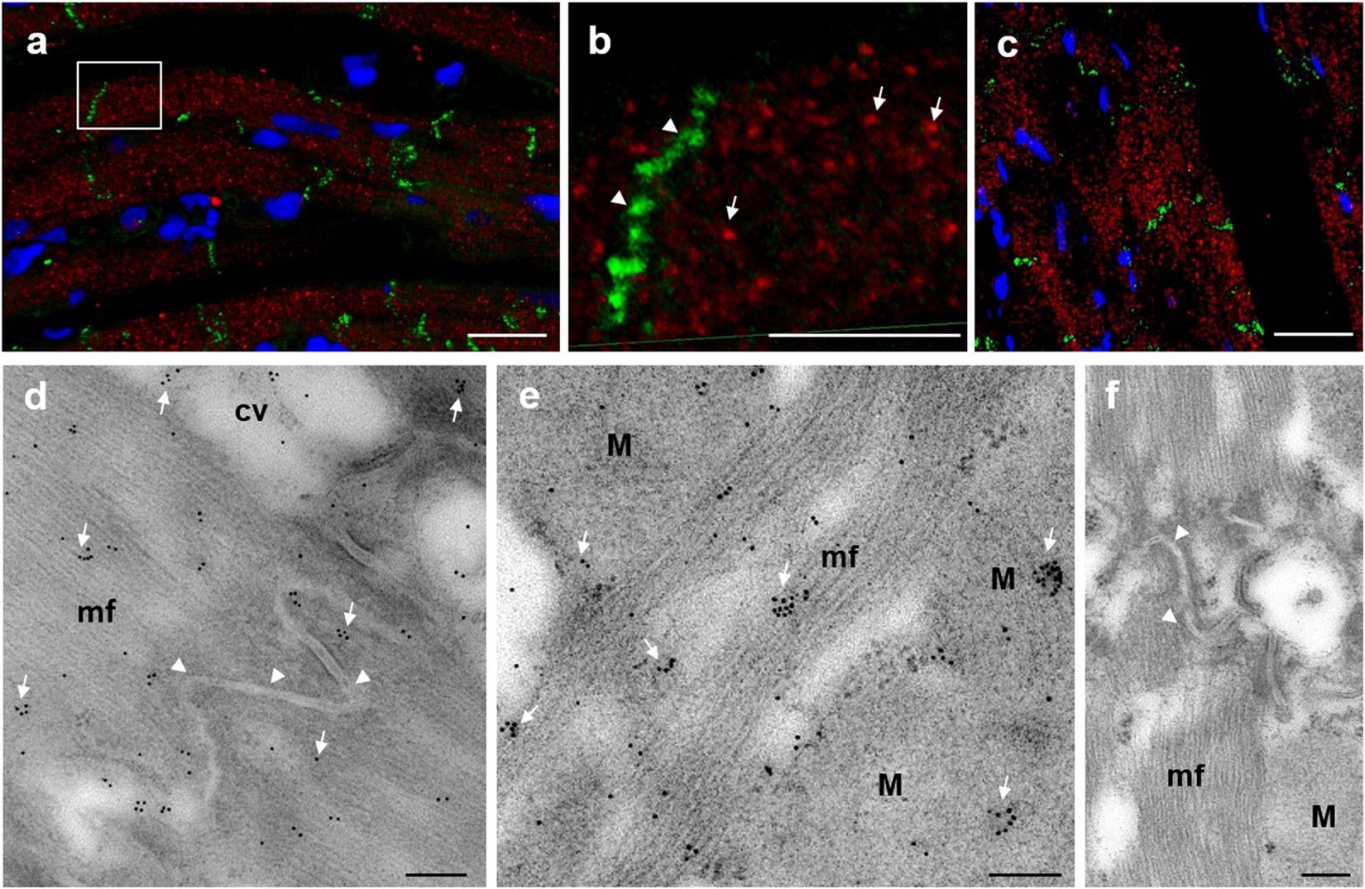
Cx26 distribution at level of the cardiomyocyte cytoplasm. (**a**–**c**) Confocal laser scanning microscopy: representative images of rat and pig heart sections. Double immunofluorescence analysis for Cx26 (red) and Cx43 (green) show a distinct cell localization of the two Cxs. (**a**) Three dimensional picture of the maximum intensity projection of the raw images of longitudinal rat heart section treated with RαCx26-cl and MαCx43. Scale bar: 20 µm. (**b**) Blow up of the square shows section view after clipping of some planes. Arrows point out the clusters of Cx26 and arrowheads intercalated discs where Cx43 is mainly localized. Scale bar: 10 µm. (**c**) Three dimensional picture of the maximum intensity projection of the raw images of longitudinal pig heart section treated with RαCx26-Ct and MαCx43. Scale bar: 50 µm. (**d**–**f**) Immuno-electron microscopy: representative pictures of immunocytochemistry for Cx26 in rat ventricle samples. Scale bars: 200 nm. (**d**) Cx26 immune-gold particles (arrows) are localized at level of myofibrils (mf) and cytoplasmic vesicles (cv) but not at the intercalated discs (arrowheads). (**e**) Cx26 gold particles (arrows) label mithocondria (M) and myofibrils (mf). (**f**) Negative control does not show Cx26 immunolabeling.

The specificity of the two primary antibodies for Cx26 was also confirmed by the reproducibility of the results. In fact, as described below, these antibodies raised against different epitopes of the antigen showed the same label site. Besides, this result was obtained in both immunoperoxidase (Fig. 3) and immunofluorescence analyses (Fig. 5a–c), as well as in immuno-electron microscopy (Fig. 5d–e).

**Figure 6:**
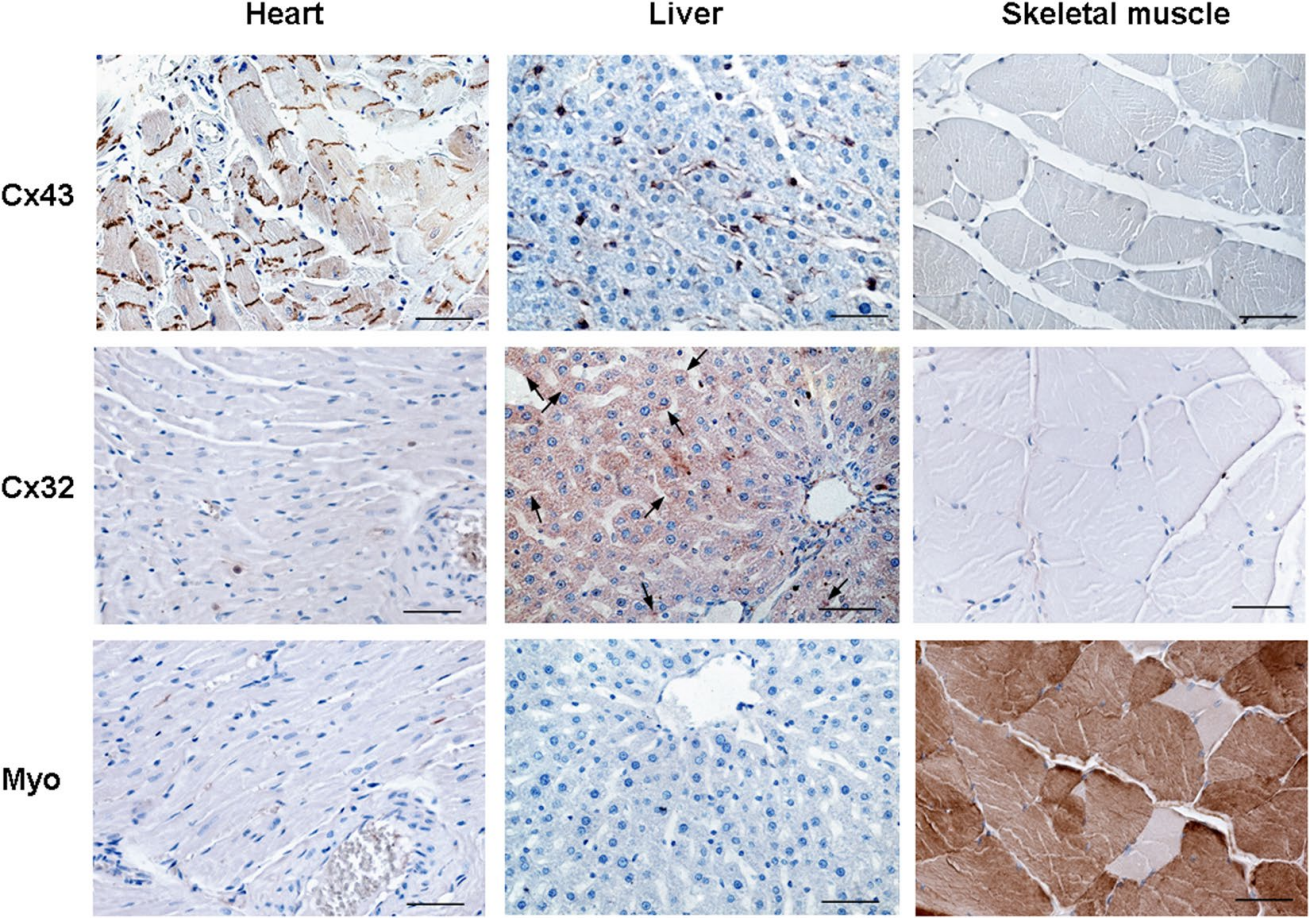
Validation of immunoperoxidase analysis on rat tissues. Representative images of immunohistochemical analysis on rat heart, liver and skeletal muscle sections by anti-Cx43, anti-Cx32 and anti-Myo antibodies. Arrow marks pointed positivity of the membrane. Scale bars: 50 µm.

Finally, both positive and negative tissue specific primary antibodies verified the validity of immunoperoxidase technique on the rat heart and on the other tissues used as controls. As shown in Fig. 6, Cx43 antibody reacted specifically with rat heart tissues at level of intercalated discs and with non-parenchymal cells of rat liver while it did not react with rat skeletal muscle. Moreover, Cx32 immunopositivity was revealed only in rat hepatocytes both on the plasma membrane and in the cytoplasm. Skeletal myosin antibody reacted only with skeletal muscle.

The results obtained by immunohistochemistry showed that both RαCx26 primary antibodies detected Cx26 in cardiomyocytes of all the analyzed heart samples and with the same pattern of positivity (Fig. 3). Cx26 was found in working cardiomyocytes of human atrium auricle, rat atrium and ventricle and in pig LV. At pig LV level, it was possible to observe the presence of Cx26 immunoreactivity also in the conduction system myocytes (asterisk in Fig. 3). Cardiomyocytes of all the species considered in this paper showed the same Cx26 cell distribution. In particular, we found the protein scattered all over the cells of pig, human and rat heart tissue sections. Interestingly, Cx26 was not observed at intercalated discs but it was distributed in the cytoplasm differently from Cx43 which was mainly localized at level of the intercalated discs (Figs 3 and 6). Double immunofluorescence confocal analysis which we performed in pig and rat cardiomyocytes confirmed the expression of Cx26 in the cytoplasm of the cells where it also appeared to be organized in clusters. Furthermore, no co-localization of Cx26 and Cx43 could be detected (Fig. 5a–c). To better identify Cx26 localization, immunogold labeling was performed on rat heart samples. As expected, positivity at intercalated discs was absent (arrowheads in Fig. 5d) while Cx26 labeling was found at level of mitochondria, myofibrils and cytoplasmic vesicles both as individual and as cluster labeling (arrows in Fig. 5d,e).

### H9c2 cells express Cx26-mRNA and -protein

**Figure 7:**
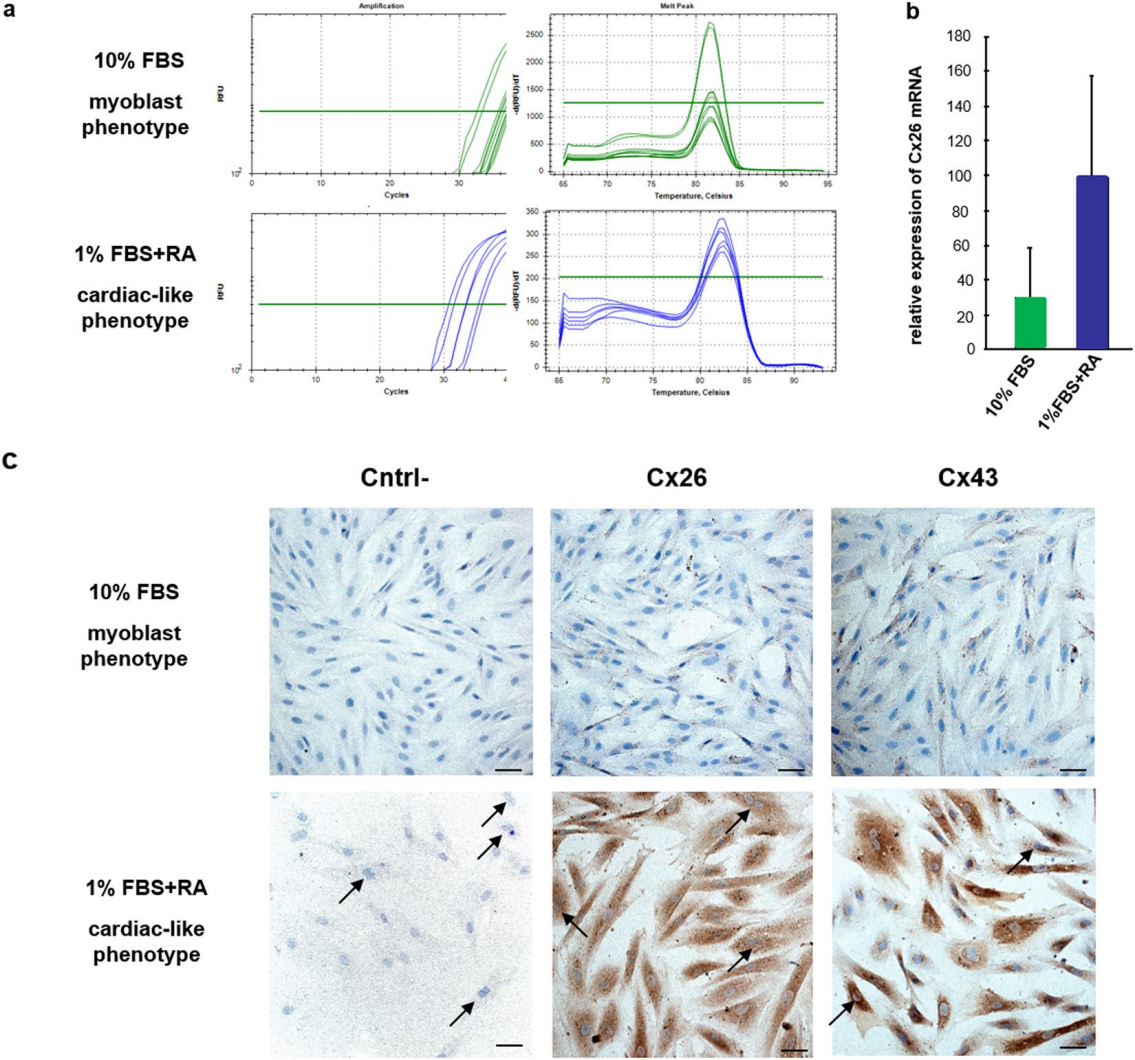
H9c2 cells express Cx26-mRNA and -protein. (**a**) Real-time PCR results obtained for Cx26 in H9c2 cell line grown with 10%FBS or with 1% FBS + 50 nM RA*. In green* threshold cycle (Ct) and relative amplification curves obtained by H9c2 cells with 10% FBS; in blue threshold cycle (Ct) and relative amplification curves obtained by H9c2 cells with 1% FBS + 50 nM RA. (**b**) Relative expression of Cx26-mRNA in H9c2 cell line grown with 10%FBS (n = 3) and with 1% FBS + 50 nM RA (n = 3). (**c**) Representative images of immunoperoxidase performed with RαCx26-cl or RαCx43 on H9c2 cells grown with 10%FBS or with 1%FBS + 50 mM RA. Arrows point out binucleated cells. Negative controls (Cntrl-) were obtained by omitting primary antibodies. Scale bars: 50 µm

To further confirm the expression of Cx26 in cardiomyocytes we performed RT-PCR (Fig. 7a,b) and immunoperoxidase analyses (Fig. 7c) on H9c2 cell line that is considered a good *in vitro* model of cardiac cells. Experiments were performed both on H9c2 myoblast-like cells and on H9c2 cells differentiated towards cardiac-like phenotype. H9c2 cardiac differentiation was confirmed by visual assessment at microscope that revealed, after RA-treatment, changes in cell morphology such as the formation of larger and multinucleated cells (arrows in the bottom of Fig. 7c) compared to myoblast H9c2 cells that showed the spindle-to-stellate shape and were commonly mononucleated (top of Fig. 7c). Moreover, differentiated H9c2 cells exhibited an increased expression of Cx43, the major connexin in the heart, compared to not-differentiated H9c2 cells (Fig. 7c).

Our results showed that H9c2 cells expressed the Cx26-mRNA (Fig. 7a) with an increasing trend in cardiac-like differentiated cells when compared to myoblast-like cells (Fig. 7b). Moreover, in the immunoperoxidase analyses H9c2 cells showed to react with the two anti-Cx26 antibodies with the same immunopositivity, which was mainly distributed at level of the cell cytoplasm near the perinuclear zone (Fig. 7c). The optical evaluation of immunoperoxidase results, obtained in triplicate tests, allowed us to clearly confirm a higher Cx26 protein expression in H9c2 cardiac-like compared to H9c2 myoblast-like cells.

## Discussion

Connexins are a family of integral membrane proteins and they are named according to their molecular weight. They share a common architecture consisting of 4 transmembrane regions, 2 extracellular loops, 1 cytosolic loop, 1 cytosolic amino terminal tail and 1 cytosolic carboxy terminal tail. The function of these proteins has been established as the major regulator of tissue homeostasis by acting at three communication levels. At intracellular level, they can interact with regulator proteins of cell proliferation and tumor progression. At the plasma membrane, six connexins can form pores, named connexons, which convey small molecules between intracellular and extracellular compartments. Moreover, connexons of adjacent cells can align to create gap junctions, allowing the exchange of small molecules between the cytoplasm of neighboring cells^13^.

Cx26 belongs to Cx protein family and has been identified in organs such as skin, ear, liver and in smooth muscle cells^6,9^. For this reason we used RαCx26 as an internal negative control in one of our previous studies on pig heart^14^. Unexpectedly, a specific immunoreaction scattered over the cytoplasm of tissue cardiomyocytes has been obtained. Therefore, we decided to perform other experiments in order to deepen this discovery. To ascertain the cardiomyocyte immunopositivity for Cx26 and to exclude possible analysis-dependent artifacts, we verified the specificity of the employed rabbit antibody (RαCx26-Ct) versus Cx26 by western blot in LV pig heart protein extracts. Moreover, we used a different primary antibody (RαCx26-cl) that was raised to a different epitope of the same Cx. It is to be noticed that these antibodies are raised against two epitopes which show the main divergence between Cx26 and other connexins at the protein level^15^. Both in western blot and in immunoperoxidase analysis RαCx26-cl antibody recognized Cx26 in pig heart.

The presence of Cx26-mRNA was investigated in pig as well as in human and rat heart samples. All samples expressed mRNA specific for Cx26. Interestingly, pig, human and rat heart samples also showed the same pattern of immunopositivity in immunoperoxidase reaction. Cx26-mRNA and -protein expression were found also in the H9c2 cell line, an established rat myocardial cell line, considered as an *in vitro* model for cardiomyocytes studies^16–18^. Most importantly, the cardiac differentiated state of the H9c2 cells showed the higher Cx26 protein and mRNA expression confirming its presence in the heart. This result is very important because it has been obtained in a cell line constituted by cardiac cells only, different from heart tissue samples where many types of cells are present in addition to cardiomyocytes.

The obtained results, together with the tissue and technical controls performed in this work, demonstrated that Cx26 is expressed in the cardiomyocytes of pig, human and rat hearts. It is to be noticed that Cx26 was not detected in heart in previous investigations^15,19^. However, this discrepancy might be due to the different technical conditions used in our work and to the very small quantity of Cx26-mRNA that we found in the healthy heart samples. Indeed, RT-PCR is much more sensitive than northern blot analysis. Moreover, paraffin-embedded immunohistochemistry may allow to reveal different epitopes from cryostatic immunohistochemistry. In all the tested samples, Cx26 showed the same pattern of cytoplasm distribution, also employing different immunohistochemistry analyses such as immunoperoxidase, immunofluorescence and immune-electron microscopy. Specifically, Cx26 was found scattered all over the cytoplasm of the cardiomyocytes. Moreover, double immunofluorescence/confocal microscopy, that allowed analyzing cardiomyocytes in three-dimensional images, showed that Cx26 was distributed in the cytoplasm, both as individual and cluster labeling, while Cx43 was mainly concentrated to the intercalated discs. Furthermore, we observed that Cx43 cytoplasmic expression did not co-label with the Cx26 cytosolic distribution. This cell distribution was confirmed with immunogold. In particular, we found Cx26 immunogold labeling at level of mitochondria, myofibrils and cytoplasmic vesicles. On the basis of these data, we could suppose that Cx26 has its own function at level of cardiomyocytes, independent from both hemichannels and gap junction makeup, considering in particular its cellular localization. Factually, Cx26 was found to be localized in the cell organelles and cytoplasm where it could interact with a variety of molecules. As reported in literature, connexins have also been identified on single or double membrane vesicles in the form of hexameric channel. These connexons accommodate different molecules that may act as a scaffold to convey information in the same cell or in the target cell^20,21^. In addition, several potential RNA- and DNA-binding motifs have been recently identified in the protein sequences of Cx26, even though the *in vivo* function of these motifs remains actually unknown^20^. Therefore, functional gap junction-independent scope of Cx26 could extend from intracellular communication to communication between nearby or more distant cells through extracellular vesicles which could transfer proteins, lipids or genetic information^22^. Already years ago, it was demonstrated that Cx26 regulates the expression of angiogenesis-related genes in human breast tumor cells^23^ or acts as a putative tumor suppressor^24,25^ by a gap junction-independent mechanism.

To our knowledge, Cx26 expression in cardiomyocytes in mammals of different species has been demonstrated for the first time in this paper. So far as we are concerned, Cx26 could be a typical mammalian cardiac protein, unlike, for example, cardiac Cx30.2 which is considered to be unique to rodents^2^. Moreover, unlike the other cardiac connexins, Cx26 did not show a spatially-dependent pattern of expression because it was found uniformly expressed by all atrial and ventricular, working and conducting cardiomyocytes.

Further detailed studies that include a better subcellular identification, as well as functional characterization of Cx26, are in progress to understand the role of this connexin in the heart and also its possible modulation in cardiac diseases. In fact, despite few heart pathological samples used in this study to examine Cx26-mRNA, it appears that its quantity increases in these samples rather than in healthy heart samples. The discovery of a new connexin expression in the heart and its possible modulation in cardiac diseases pave the way for future investigations focused on the physiopathological role of Cx26 in the heart.

## Materials and Methods

### Tissue samples

Human heart tissue samples were collected from human right atrium (auricle) of valvular heart patients (n = 2) with left ventricular ejection fraction (LVEF) % > 50% at the moment of surgical procedures for resolution of valvular defects and from ventricle (n = 4) of end-stage heart failure patients undergoing to assist device implantation (VAD). The research conforms to the principles outlined in the Declaration of Helsinki (Br Med J 1964; ii:177). It was approved by the Ethics Committee of the Niguarda Ca’ Granda Hospital of Milan and all patients provided signed informed consent.

Rat tissue samples were collected from 12-week-old male Wistar albino rats (n = 3) (Charles River, Calco, LC, Italy). The animals, treated in accordance with the European Communities Council Directive of 24 November 1986 (86/609/EEC and 2010/63/UE), in agreement with the Italian DM26/14, were allowed unrestricted access to food and water. Rats were euthanized by a lethal dose of chloral hydrate (Sigma-Aldrich, St. Louis, MO, USA), and their hearts, livers and quadriceps muscles were dissected. The experimental protocol was approved by the Animal Care and Use Committee of the University of Pisa and was in compliance with the National and European guidelines for handling and use of experimental animals.

Pig heart left ventricle (LV) tissue samples were collected from healthy male adult minipigs (n = 6). Left intercostal thoracotomy was performed under general anesthesia and sterile conditions. These procedures were approved by the Animal care Committee of the Italian Ministry of Health, were in conformity with the Guide for the Care and Use of Laboratory Animals published by the US National Institutes of Health (NIH Pubblication No. 85-23, revised 1996) and were performed at the Experimental Biomedicine Centre of G. Monasterio Foundation.

Collected tissue samples were washed in PBS and immediately placed in RNAlater (Sigma Aldrich, Italy) or lysis buffer or 4% paraformaldehyde for biomolecular analysis, Real Time-PCR or western blotting, or paraffin embedding/immunohistochemistry analysis. Some ventricle rat samples were collected for immunogold analysis.

### Rat cardiomyoblast cell line H9c2

The H9c2 myogenic cell line, derived from embryonic rat heart myocardium (American Tissue Culture Collection, Manasas, VA, USA), is considered a suitable *in vitro* model for heart studies^17,18^. This cell line was maintained at 37 °C-5% CO_2_ humidified atmosphere in Dulbecco’s modified Eagle’s Medium (DMEM; Sigma-Aldrich) with 10% fetal bovine serum (FBS; Sigma-Aldrich) and antibiotics (25 U/ml penicillin and streptomycin, Sigma-Aldrich). The cardiac differentiation was performed by serum reduction to 1% and all trans-retinoic acid (RA; Sigma-Aldrich) supplementation (50 nM) for 10 days replacing medium every 3 days^18,26^. The cells earmarked for immunohistochemistry were grown (5,000/spot) on slides and then fixed in 1% formalin/PBS for 15 min at 4 °C; the cells earmarked for Real Time-PCR were treated on plastic plates, trypsinized and re-suspended in acid guanidinium thiocyanate-phenol-chloroform (about 350 µl per 200,000–500,000 cells of TRI-Reagent, Lonza, Switzerland, CH).

### mRNA extraction, cDNA synthesis, primer design and Real-Time PCR experiments

The total mRNA was extracted from each sample using an Rneasy Mini kit (Qiagen S.p.A, Milano, Italy) following a standardized method^27^ and then stored at −80 °C until used. The mRNA concentration and purity were determined spectrophotometrically (BioPhotometer, Eppendorf Italy, MI) measuring spectral absorption at 260 nm. The reading ratio at 260 nm and 280 nm (A_260_/A_280_) provided an estimate of RNA purity with respect to contaminants absorbed in the UV spectrum, such as protein. Only RNA samples with spectrophotometric OD 260/280 ratios of 1.9–2.1 were used. The RNA samples were stored at −80 °C to be used in gene expression studies.

First strand cDNA was synthesized with iScript cDNA Synthesis kit (Bio-Rad, Hercules, CA, USA) using about 0.5-1 µg of total RNA as a template. Reverse transcriptase reaction sequence consisted of incubation at 25 °C for 5 min, followed by three different cycles at 42 °C for 30 min, at 45 °C and 48 °C for 10 min, in order to better separate the strands. The reverse transcriptase enzyme was inactivated by heating at 85 °C for 5 min. The cDNA samples obtained were placed on ice and stored at 4 °C until further *use*. Specific human and rat primers for Cx26 were designed by Primer Express Version 2.0 (Applied Biosystems) and synthesized by Sigma-Aldrich; whenever possible, intron-spanning primers or exon-exon primers were selected to avoid amplification of genomic DNA (Table 1). Reaction conditions for each Cx26 primer were optimized; i.e., a gradient PCR was conducted to assess the optimal annealing temperature, while a standard curve obtained by scalar dilution of a cDNA pool (1:5, 1:25, 1:125, 1:625) was always generated to verify PCR efficiency (optimal value obtained in the range of 95–105% and a linear standard curve, R^2^, greater than |0.990|). Cx26 mRNA transcriptomic profile was assessed by Real-Time PCR studies. The reactions were performed in duplicate in the Bio-Rad C1000™ thermal cycler (CFX-96 Real-Time PCR detection systems, Bio-Rad Laboratories Inc., Hercules, CA, USA). To monitor cDNA amplification, a third-generation fluorophore, EvaGreen, was used (SsoFAST EvaGreen Supermix, Bio-Rad). PCR was performed in a volume of 20 μl per reaction, including 0.2 μM of each primer (Sigma-Aldrich) sample, reagent and sterile H_2_O. Amplification protocol started with 98 °C for 30 s followed by 40 cycles at 95 °C for 5 s and 60 °C for 30 s. To assess product specificity, amplicons were checked by melting curve analysis. Melting curves were generated from 65 °C to 95 °C with increments of 0.3 °C/cycle.

**Table 1:** Details of primers for the evaluated genes.

*Genes*	*Species*	*Sequences*	*GenBank, accession n°*	*bp*	*Ta (°C)*
*Cx26*	Pig	**F:** GAAGAGCGAGTTCAAGGA **R:** AAGAGGATGCGGAAGA	XM_021065430	101	60
*Cx26*	Human	**F:** CCACAGAGGACACAGAGAA **R:** CCAAAGCAAATGAAAGAACCAAT	NM_004004	99	60
*Cx26*	Rat	**F:** GCTCACTGTCCTCTTCATC **R:** AATCGGCTTGCTCATCTC	NM_001004099	80	60
*UBC*	Rat	**F:** ATCTAGAAAGAGCCCTTCTTGTGC **R:** ACACCTCCCCATCAAACCC	NM_17314.1	50	60
*YWHAG*	Rat	**F:** TTCCTAAAGCCCTTCAAGGCA **R:** GGCTTTCTGCACTAGTTGCTCG	NM_019376.2	100	60
*PPIA*	Rat	**F:** CCAAACACAAATGGTT **R:** ATTCCTGGACCCAAAACGCT	NM_017101	100	60

***Ta:*** annealing temperature. **UBC:** ubiquitin, **YWHAG:** tyrosine 3-monooxygenase7tryptophan 5-monooxygenase activation protein, gamma polypeptide, **PPIA:** peptidylprolyl isomerase a (cyclophilin a).

Relative levels of Cx26-mRNA were detected in H9c2 cell line. In particular, we evaluated Cx26 mRNA expression in H9c2 cell line grown with 10%FBS (n = 3) and with 1% FBS + 50 nM RA (n = 3). The relative expression was obtain normalizing the Real-Time PCR results with the geometric mean previously tested^26^ of the three most stably expressed genes (UBC, YWAG, PPIA) (Tab.1) following the MIQE (Minimum Information for publication of Quantitative Real-Time PCR Experiments) guidelines^28^ (Bustin *et al*., 2009 Clinical Chemistry 55, 611–622). The relative quantification was performed by ΔΔCt method using Bio-Rad’s CFX96 manager software (CFX-96 Real-Time PCR detection systems, Bio-Rad Laboratories Inc.).

### Primary antibodies

Two different rabbit primary antibodies were used to detect Cx26, one raised against a peptide near the C-terminus of human origin (RαCx26-Ct, sc-130729, Santa Cruz Biotechnology, Santa Cruz, CA, USA) and the other raised against a segment of cytoplasmic loop of human Cx26 (RαCx26-cl, NBP2-41304, Novus Biologicals, Abingdon, UK). Cx43 was detected by using two primary antibodies raised against a peptide near the C-terminus of human origin, one obtained in rabbit (RαCx43, NB1000-91717, Novus Biologicals) and the other, used for double immunofluorescence, in mouse (MαCx43, sc59949, Santa Cruz Biotechnology)^14^. Rabbit anti-Cx32 antibody raised against a portion of the cytoplasmic loop of rat Cx32 was purchased from Thermo Scientific (71-0600, Waltham, Massachusetts, USA). Mouse anti-skeletal myosin antibody was purchased from Sigma-Aldrich (Myo, M4276). Rabbit anti-GAPDH was purchased from Sigma Aldrich (G9545).

### Western blotting

Tissue lysates was obtained incubating small fragments of tissues in lysis buffer (50 mM Tris-HCl, pH 7.4, 150 mM NaCl, 1 mM EDTA, 1 mM EGTA, 1% NP-40, 0.1% SDS, 0.5% sodium deoxycholate, 1 mM Phenylmethylsulfonyl fluoride) which contained serine/threonine and tyrosine phosphatase inhibitors and a protease inhibitor cocktail 1× (S8830, Sigma Aldrich). Then the samples were homogenized by using two 10 sec pulses by Micro-Ultrasonic Cell Disrupter (Vineland, NJ, USA) set at maximum output in an ice bath and centrifuged at 15,000 rpm for 20 min at 4 °C. Supernatant protein concentration was determined by the BCA microplate method (Thermo Fisher). Proteins (60 µg/lane) were separated on a 4–20% polyacrylamide gel (BioRad, Hercules, CA, USA) under reducing conditions and transferred to a nitrocellulose membrane (Trans Turbo Blot system, BioRad). The membrane was blocked with 4% dry fat milk in TBS Tween 0.1% (T-TBS) and incubated (overnight at 4 °C) with RαCx26-Ct or RαCx26-cl diluted 1:500 and 1:1000, respectively in T-TBS. Rabbit anti-GAPDH antibody diluted 1:10000 in T-TBS was used as protein loading control. Anti-rabbit HRP-conjugated antibodies (BioRad) (1:2000 in 4% dry fat milk in T-TBS, 1 h) were used as secondary antibodies and immunocomplexes were detected by chemiluminescence (ECL clarity, BioRad), using Chemi-Doc XR (BioRad). Negative controls were performed omitting primary antibodies to ascertain secondary antibodies unspecific binding. All reactions were performed at room temperature unless otherwise specified. Image lab software (BioRad) was used to evaluate both the right molecular weights of the resulting bands and their density.

### Immunohistochemistry

5–8 µm-thick sections were collected from paraffin-embedded tissue samples and mounted on slides. Before use, slides were dewaxed, rehydrated and processed for immunoperoxidase or immunofluorescence analysis. Reactions were performed in humid chambers at room temperature, unless otherwise specified. For each sample, at least 3 non-consecutive sections were examined.

#### Immunoperoxidase analysis

Regarding the antigen retrieval, tissue sections were exposed to microwaves for 15 min at 600 W in 10 mM sodium citrate while H9c2 cells were treated with 0.2%, triton-X100/PBS (Sigma-Aldrich) for 10 min. Then the samples were incubated for 10 min in 3% H_2_O_2_/methanol in order to block endogenous peroxidases, treated for 20 min at 37 °C with PBS-blocking solution containing 1% normal goat serum (NGS, Vector, Burlingame, CA, USA), 0.1% bovine serum albumin (BSA, Sigma-Aldrich), 0.1% triton-X100 for blocking non-specific binding. and then incubated overnight at 4 °C with primary antibodies diluted in a 1%BSA/2%FBS/PBS solution (RαCx26-Ct 1:400, RαCx26-cl 1:200, RαCx43 1:200, RαCx32 1:200 and MαMyo 1:750). Detection was accomplished by sequential treatments with biotinylated anti-rabbit or anti-mouse immunoglobulins (1:200, BA-1100 or BA-2000, Vector), streptavidin-peroxidase complex (Vector) and 3.3’diaminobenzidine tetrahydro-chloride (DAB Dako Italia SRL, Milan, Italy). The specificity of secondary antibodies was obtained by performing the experiments omitting the primary antibodies. Sections were then counterstained with Harris’ hematoxylin (Fluka, Buchs, Switzerland) and examined by means of a Leica DMRB light microscope at 20x or 40x magnification; representative images were captured by DFC480 digital camera (Leica Microsystem, Cambridge, UK).

#### Double-immunofluorescence analysis

Slides with tissue sections were treated for 10 min with 0.2% triton-X100/PBS and, after 1 hour in blocking solution (0.1% Tween, 0.25% BSA in PBS), they were incubated overnight at 4 °C with primary antibodies diluted in blocking solution as follows: RαCx26-Ct 1:200 and MαCx43 1:100, RαCx26-cl 1:100 and MαCx43 1:100. Slides were then washed three times in blocking solution and incubated for 90 min in the dark with relative fluorescent secondary antibodies diluted 1:250 in blocking solution (Alexa Fluor® 488 anti-mouse and Alexa Fluor® 568 anti-rabbit, Life Technologies Italia, Monza, MB, Italia). Nuclear staining was performed incubating the slides with 1 µM TO-PRO (TO-PRO®-3stain, Life Technologies Italia) for 15 min in the dark. Samples were mounted with PBS-glycerol solution. All steps were performed at room temperature unless otherwise specified. The samples were observed with a confocal laser scanning microscope (TC SSP8 Leica Microsystems, Mannheim, Germany) using a 488-nm, 561-nm and 642-nm excitation wavelength lasers.

#### Immuno-electron microscopy

Specimens of rat myocardium tissue were fixed in a solution containing 2% paraformaldehyde and 0.1% glutaraldehyde in 0.1 M PBS (pH 7.4) for 90 min at 4 °C and postfixed in 1% osmium tetroxide (OsO_4_) in the same buffer for 1 h at 4 °C. This method, which combines aldehyde and mild OsO_4_, allows a minimal cover of antigen epitopes while preserving cell architecture and sub-cellular structures^29^. Specimens were then dehydrated in ethanol and embedded in epoxy resin. Ultrathin sections (60–80 nm) obtained with a Reichert-Jung Ultracut E, equipped with a diamond knife, were collected on nickel grids and incubated in an aqueous satured sodium metaperiodate at room temperature, as previously described^29,30^. To block non-specific antigenic sites, nickel grids were incubated in a cold PBS-blocking solution containing 10% normal goat serum and 0.2% saponin for 20 min. Then, grids were incubated with RαCx26-cl (1:50 in 1% goat serum/0.2% saponin/PBS) overnight at 4 °C, rinse in PBS and then incubated with a 10 nm-gold-conjugated secondary antibody (1:20 in 1% goat serum/0.2% saponin/PBS; 15726, Ted Pella Inc, Redding, CA, USA) for 1 h. After rinsings in PBS, grids were incubated with 1% gluteraldehyde for 3 min, washed in distilled water, counterstained with uranyl acetate and lead citrate and observed under a Jeol JEM100 SX transmission electron microscope. Control sections were obtained by omitting the primary antibody and by incubating with the secondary antibody only.

### Statistical analysis

Relative quantification of Cx26 was calculated by the ΔΔCt method using Bio-Rad’s CFX96 manager software. Differences between two independent groups were assessed by unpaired t-test. The results are expressed as mean ± SEM and p-value was considered significant when < 0.05.

## Data Availability

The data sets generated during the current study are available from the corresponding author on reasonable request.
